# Nanoparticle fouling and its combination with organic fouling during forward osmosis process for silver nanoparticles removal from simulated wastewater

**DOI:** 10.1038/srep25859

**Published:** 2016-05-10

**Authors:** Yanxiao Zhao, Xinhua Wang, Zhiwei Wang, Xiufen Li, Yueping Ren

**Affiliations:** 1 Jiangsu Key Laboratory of Anaerobic Biotechnology, School of Environmental and Civil Engineering, Jiangnan University, Wuxi 214122, P. R. China; 2 State Key Laboratory of Pollution Control and Resource Reuse, School of Environmental Science and Engineering, Tongji University, Shanghai 200092, P. R. China

## Abstract

The increasing and wide application of silver nanoparticles (Ag NPs) has resulted in their appearance in wastewater. In consideration of their potential toxicity and environmental impacts, it is necessary to find effective technology for their removal from wastewater. Here, forward osmosis (FO) membrane was applied for Ag NPs removal from wastewater, and single and combined fouling of nanoparticles and organic macromolecules were further investigated during the FO process. The findings demonstrated that FO membrane can effectively remove Ag NPs from wastewater due to its high rejection performance. Fouling tests indicated that water flux declined appreciably even at the beginning of the single Ag NPs fouling test, and more remarkable flux decline and larger amounts of deposited Ag NPs were observed with an increase of Ag NPs concentration. However, the addition of bovine serum albumin (BSA) could effectively alleviate the FO membrane fouling induced by Ag NPs. The interaction between Ag NPs and BSA was responsible for this phenomenon. BSA can easily form a nanoparticle-protein corona surrounded nanoparticles, which prevented nanoparticles from aggregation due to the steric stabilization mechanism. Furthermore, the interaction between BSA and Ag NPs occurred not only in wastewater but also on FO membrane surface.

In the past decades, nanoparticles have attracted much attention due to the increase of production and application for their unique physicochemical properties and their potential threats to the natural environment^1^,^2^,^3^. As one of the most promising metals of various nanoparticles with the properties of antiviral and antimicrobial, silver nanoparticles (Ag NPs) have been widely used in the daily life including textiles, cosmetics, household products and wound dressing^4^. Ag NPs applied in many consumer products can result in transfer to the aqueous environment via wastewater treatment plants (WWTPs)^5^,^6^. In consideration of the potential hazard of Ag NPs to aqueous ecosystem and their long-term effect on human health^1^,^3^,^7^,^8^,^9^,^10^, it is necessary to explore effective way to remove Ag NPs from wastewater.

Recently, forward osmosis (FO) membrane and its combination with biological technologies such as osmotic membrane bioreactors (OMBRs) have attracted growing interests in sea/brackish water desalination and wastewater reclamation due to its high rejection, low fouling tendency and low energy requirement^11^,^12^,^13^,^14^,^15^,^16^,^17^. As a non-porous osmotically-driven membrane, FO membrane has high rejection for pollutants in wastewater^18^. Based on this fact, some researchers have pointed out that FO membrane can be used for nanoparticles removal from wastewater^18^. During the process of nanoparticles removal, FO membrane will inevitably encounter the membrane fouling induced by nanoparticles and their combination with organic matters in wastewater.

FO is generally considered to a low-fouling membrane process due to its lack of hydraulic pressure and lower flux conditions^19^; however, an FO can be still subject to the risk of particle deposition, biofilm formation and scaling under unfavorable conditions^20^,^21^. The formed fouling layer during the FO process can lead to the flux decline due to the increase of the hydraulic resistance of the fouled membrane and/or the cake enhanced osmotic pressure (CEOP) effect^22^. According to the specific type of foulants, FO membrane fouling can be generally categorized into biological fouling, inorganic scaling, organic fouling, and colloidal/particulate fouling^23^,^24^,^25^,^26^. Considering their properties, the Ag NPs fouling belongs to colloidal/particulate fouling of FO membrane according to the classification of FO membrane fouling. With regard to organic fouling and its combination with inorganic or colloidal fouling, comprehensive insights have been given by previous studies^21^,^23^,^25^,^27^,^28^,^29^,^30^. The organic (e.g., natural organic matters, polysaccharides, proteins) fouling behavior of FO membranes was investigated by a series of typical model compounds such as humid acid, sodium alginate and bovine serum albumin (BSA)^23^,^25^,^27^. Furthermore, several researches intend to analyze the combined organic and colloidal fouling through changing solution chemistries (pH and ion concentrations) and applying hydraulic pressure on the feed side^25^,^29^. However, in general, only colloidal silica is applied in all studies on the colloidal/particle fouling and its combination with organic fouling during FO membrane process^25^,^29^. Thus, it is necessary to investigate the nanoparticle fouling and its combination with organic fouling in order to better understand the membrane fouling mechanisms during FO process.

This study aims to evaluate the feasibility of Ag NPs removal from wastewater by FO membrane, and to investigate the fouling behaviors of single Ag NPs and their combination with BSA fouling in the FO process. BSA is chosen as the model organic matters based on the fact that proteins are common organic matters during wastewater treatment. To the best knowledge of the authors, this is the first study for analyzing the fouling behaviors of Ag NPs in the FO process.

## Results and Discussion

### Process performance

In order to identify the Ag NPs removal and fouling behaviors of Ag NPs and their combination with BSA, FO experiments with feed Ag NPs concentrations of 1 and 10 mg/L, BSA concentration of 200 mg/L and their combinations were performed according to the fouling test procedures mentioned above. In all FO experiments with Ag NPs in the feed wastewater, the Ag NPs can not be detected in the draw solution. It demonstrated that FO membrane could efficiently retain the Ag NPs due to its high rejection. Compared to the traditional technologies for nanoparticles removal such as coagulation^31^,^32^,^33^, FO membrane can achieve higher removal efficiency. The concentration factor (*CF*_*w*_) during the FO process were 2.62, 2.29, 2.89, 2.84 and 2.75 for the feed solution of 1 mg/L Ag NPs, 10 mg/L Ag NPs, 200 mg/L BSA, 1 mg/L Ag NPs & 200 mg/L BSA and 10 mg/L Ag NPs & 200 mg/L BSA, respectively. It means that the residual wastewater enriched in the AgNPs. The concentrated AgNPs can be transformed into Ag_2_S, and then the potential disposal is landfill based on the fact that Ag_2_S is stable during the treatment^34^.

**Figure 1:**
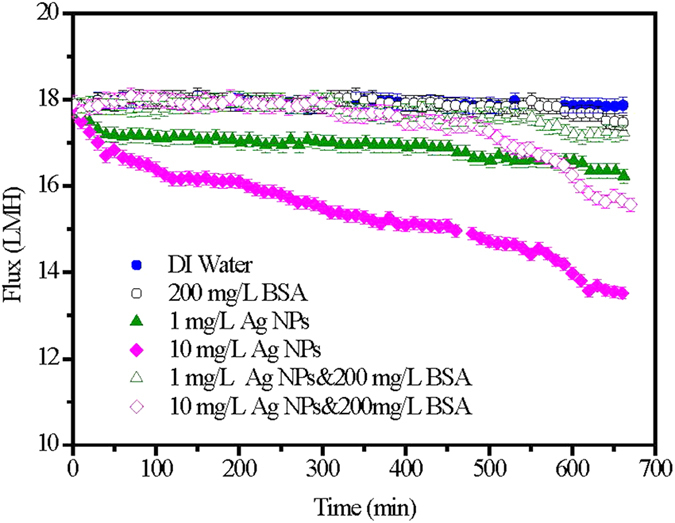
The flux curves of different combinations of Ag NPs and BSA during the fouling tests. Experimental conditions were as follows: thin film composite (TFC) FO membranes at the mode of AL-FS, 5.0 M NaNO_3_ as draw solution, temperatures controlled at 25 ± 1 °C and cross-flow rate of 12.9 cm/s.

Figure 1 presents the result of water flux variations during the FO membrane fouling tests with 1 mg/L and 10 mg/L Ag NPs, 200 mg/L BSA and their combinations as the feed solution. As shown in Fig. 1, with regard to the single BSA as the feed solution, there was no flux decline during the whole test. However, as for the single Ag NPs as the feed solution, significant flux decline could be observed at both Ag NPs concentrations. According to their variations of water flux during the fouling test, the flux decline of both Ag NPs could be divided into two stages. The first stage was located in the first 50 min, while the other operating time belonged to stage 2. During stage 1, the flux declined appreciably at both Ag NPs concentrations. Such a significant flux drop is most likely due to the Ag NPs aggregation by random Brownian diffusion that can be easily deposited on the membrane surface, and in the meantime, the nanometer diameter enables them to easily block the membrane pore^35^,^36^. Compared to the flux decline of 1 mg/L Ag NPs, the flux decline of 10 mg/L Ag NPs was more serious in stage 1. With regard to stage 2, the flux decline induced by 10 mg/L Ag NPs continued decreasing until the end of fouling experiment, while the flux curve of 1 mg/L Ag NPs became stable and just had a slight decline at the end. The sharp permeate flux loss caused by large concentrations of nanoparticles might be attributed to CEOP, which severely reduces effective osmotic pressure induced by the high concentration of salt at the membrane surface^37^. The severer flux decline of 10 mg/L Ag NPs at both stages compared to 1 mg/L Ag NPs suggested that the FO membrane fouling might be deteriorated with the increase of Ag NPs concentrations.

With regard to the combined foulants of BSA and Ag NPs, the flux drop was more serious than that of single BSA but much less than that of single Ag NPs regardless of the concentration. It means that the addition of BSA could mitigate the FO membrane fouling induced by Ag NPs, while the Ag NPs could deteriorate the BSA fouling of FO membrane. Further analyses of the variations of flux decline showed the significant difference between the BSA and Ag NPs of 1 mg/L and BSA and Ag NPs of 10 mg/L. Before 300 min, the flux curve of BSA and Ag NPs with low concentration was almost the same as that with high concentration. However, after 300 mins, the flux significantly dropped for BSA and Ag NPs with high concentration, while it was only slightly declined for BSA and Ag NPs with low concentration. As a whole, the flux decline of BSA and Ag NPs with high concentration was more serious.

### Fouling observation of fouled FO membranes by Ag NPs and their combination with BSA

**Figure 2:**
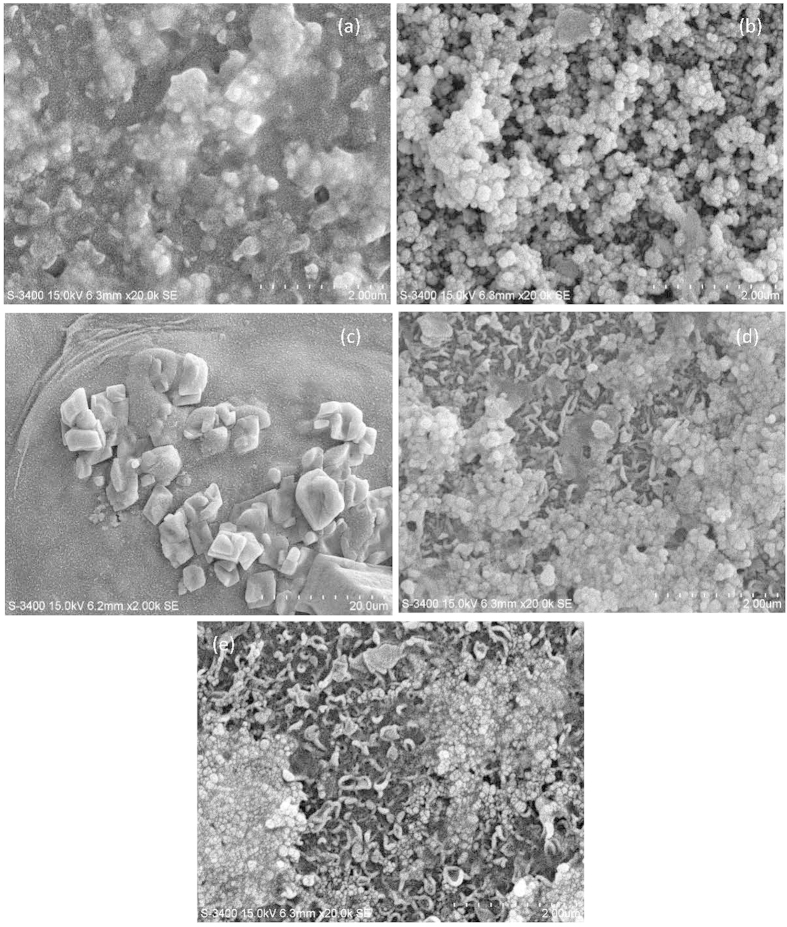
SEM images of fouled FO membranes under different feed solutions. (**a**) 1 mg/L Ag NPs; (**b**) 10 mg/L Ag NPs; (**c**) 200 mg/L BSA; (**d**) 1 mg/L Ag NPs and 200 mg/L BSA; (**e**) 10 mg/L Ag NPs and 200 mg/L BSA.

After the fouling tests on the single foulants of Ag NPs and BSA and their combinations, the fouled FO membranes were analyzed by scanning electron microscope (SEM) and confocal laser scanning microscope (CLSM) using fluorescein isothiocyanate (FITC) as the staining dye. The surface images of all fouled FO membranes by Ag NPs and BSA are shown in Fig. 2. With regard to the fouled FO membrane induced by low concentration of Ag NPs (Fig. 2(a)), its surface was deposited by Ag NPs with relatively large size mostly due to the aggregation and the sodium dodecylbenzene sulfonate (SDBS) used as a dispersant in the feed solution. However, as for the high concentration of Ag NPs (Fig. 2(b)), the fouled FO membrane surface was covered with more particles but less SDBS. Furthermore, much larger, clumpy and angulate foulants could be found in the fouled FO membrane surface with the BSA (Fig. 2(c)) as the feed solution, whose fouling behavior was significant different from the Ag NPs. It is interesting to note that after the addition of BSA, the Ag NPs fouling on membrane distributed unevenly, and the membrane surface could be found in some regions (Fig. 2(d,e)). Furthermore, the particle size seemed to become smaller on the FO membrane with the combined foulants than that only with Ag NPs in the feed solution. These observations suggested that the addition of BSA might play a significant role in Ag NPs aggregation and fouling on FO membrane surface.

**Figure 3:**
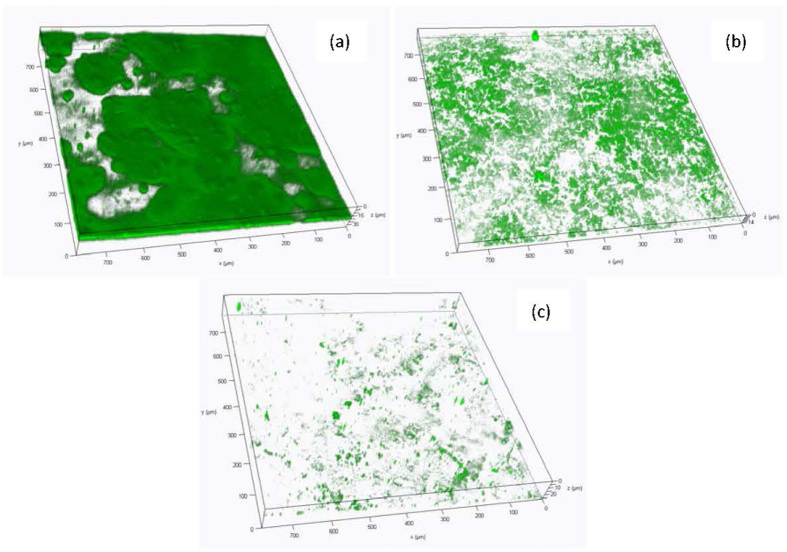
CLSM images of proteins stained with FITC in the FO fouling layer formed by different foulants. (**a**) 200 mg/L BSA; (**b**) 200 mg/L BSA and 1 mg/L Ag NPs; (**c**) 200 mg/L BSA and 10 mg/L Ag NPs.

The CLSM images of fouled FO membranes with the foulants of BSA and the combinations of BSA and Ag NPs are summarized in Fig. 3. It could be seen from Fig. 3(a) that BSA in the fouling layer was distributed in dispersed form and aggregated into some thick clusters. However, when the combinations of BSA and Ag NPs as the foulants, the morphology and structure of fouling layer were significantly changed. As shown in Fig. 3(b), after 1 mg/L of Ag NPs was added into the BSA solution, the thickness of the fouling layer formed by proteins decreased, and the BSA became more scattered. When the concentration of Ag NPs was further increased to 10 mg/L, the thickness of the fouling layer was increased due to a large amounts of Ag NPs deposited on membrane surface^37^; however, the BSA became less abundant and appeared only in some spots. It might be attributed to the fact that the Ag NPs can destroy part of the structure of the protein as a result of surface adsorption^38^. With an increase of Ag NPs concentration, the amounts of BSA adsorbed on the surfaces of Ag NPs increased, and thus the free proteins correspondingly reduced.

### Foulant compositions of fouled FO membranes by Ag NPs and their combination with BSA

**Figure 4:**
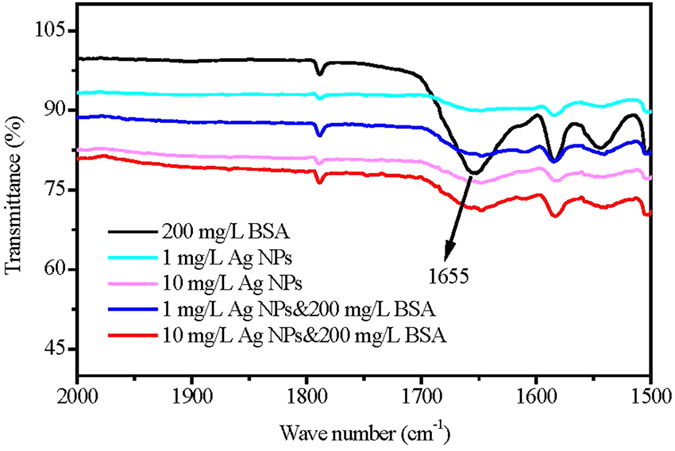
The FTIR spectrums of fouled membrane surfaces induced by single and combined BSA and Ag NPs during the fouling tests.

Attenuated total reflection-Fourier transform infrared spectroscopy (ATR-FTIR) was used to characterize the composition of proteins on the fouled membrane surfaces by using the sensitivity of amide bands to peptide structure^39^. There is a correlation between the frequency (absorption of amide I and II of a protein) and the secondary structure of proteins. The stretching vibration frequency of amine I depends on hydrogen bonding properties between C=O and NH, namely the characteristics of the vibration frequency reflects the specific secondary structure of proteins or peptides^40^. The absorption occurs in the region 1652–1657 cm^−1^ (amide I) for the proteins having mainly α-helical structure in an aqueous solution^41^. Figure 4 shows the FTIR spectrums of proteins on the fouled membrane surfaces by single and combined BSA and Ag NPs during the fouling tests. A sharp adsorption peak at 1654 cm^−1^ that is characteristics to protein secondary structures only occurred on single 200 mg/L BSA fouled membrane surface, while the peak diminished after the addition of different concentrations of Ag NPs. It indicated that the addition of Ag NPs can destroy the α-helical secondary structure of the protein.

### Combined fouling behaviors of BSA and Ag NPs

**Figure 5:**
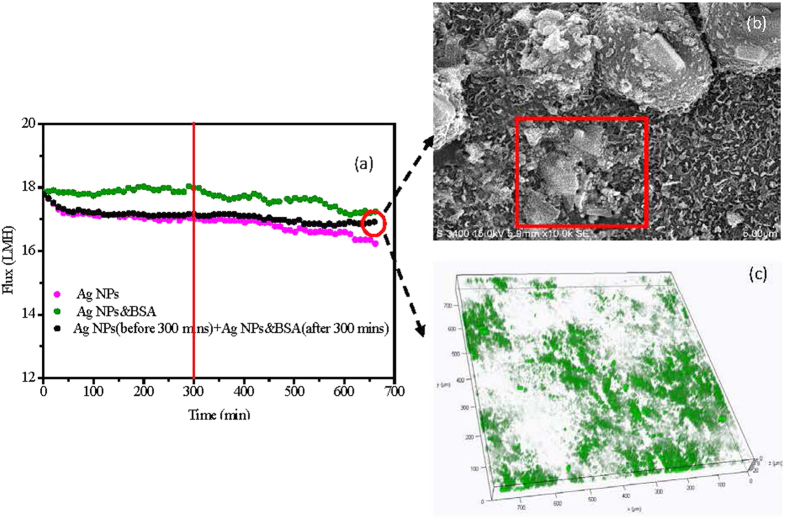
Combined fouling behaviors of BSA on Ag NPs. (**a**) Flux curve during the combined fouling; (**b**) SEM image of the fouled FO membrane; (**c**) CLSM image of the fouled FO membrane.

According to Fig. 1, the operating time of 300 min was a critical point of the combined fouling behaviors of BSA and Ag NPs, before which the water flux was stable and not influenced by the increase of Ag NPs, but after which the flux decline was observed and significantly affected by the concentration of Ag NPs. Thus, in order to further identify the combined fouling behaviors of BSA and Ag NPs, further investigation on the fouling behavior by single Ag NPs before 300 min and then the combination of BSA and Ag NPs was conducted. Specifically, 1 mg/L Ag NPs fouling experiment was continuously performed with freshly prepared feed solution and draw solution for 300 mins to form an Ag NPs fouling layer on membrane surface, and then immediately adding 200 mg/L BSA into the feed solution for other 360 mins operations. As shown in Fig. 5(a), the addition of BSA after Ag NPs fouling for 300 mins resulted in a slight flux decline over the rest fouling experiments. It could also be observed that its flux value was higher than that of the FO membrane used only Ag NPs as the foulant in the feed solution, suggesting that the addition of BSA after 300 mins could also alleviate the FO membrane fouling induced by Ag NPs. However, the mitigation of Ag NPs fouling with the addition of BSA after 300 mins was worse than the addition of BSA in the whole process. It implied that the addition of BSA played a more significant role in mitigating the Ag NPs fouling before the fouling layer formed although the BSA could further alleviate the Ag NPs fouling after the fouling layer formed. The SEM and CLSM observations were also performed for the fouled FO membrane with single Ag NPs initially and then the combination of BSA and Ag NPs as the foulants. As shown in Fig. 5(b), the continuous particle layer was broken compared to the single Ag NPs fouling as shown in Fig. 2(a), but the size of the aggregation of Ag NPs seemed to be larger than that of the addition of BSA during the whole process as shown in Fig. 2(d). The CLSM image exhibited that the deposited BSA was more, and the fouling thickness layer was higher compared to the addition of BSA during the whole process as shown in Fig. 3(b). These results were consisted with the variations of water flux shown in Fig. 5(a). According to the report from Gu *et al.*^42^, the FO membrane surface property was responsible for the organic fouling before the fouling layer formed; however, foulant-foulant interaction instead of membrane surface property played a dominant role in FO membrane fouling before the fouling layer formed. Based on the results mentioned above, it could be concluded that not only the interactions of Ag NPs and the FO membrane surface but also the foulant-foulant interactions were influenced by the addition of the BSA.

### Mechanisms for the combined Ag NPs and BSA fouling

**Figure 6:**
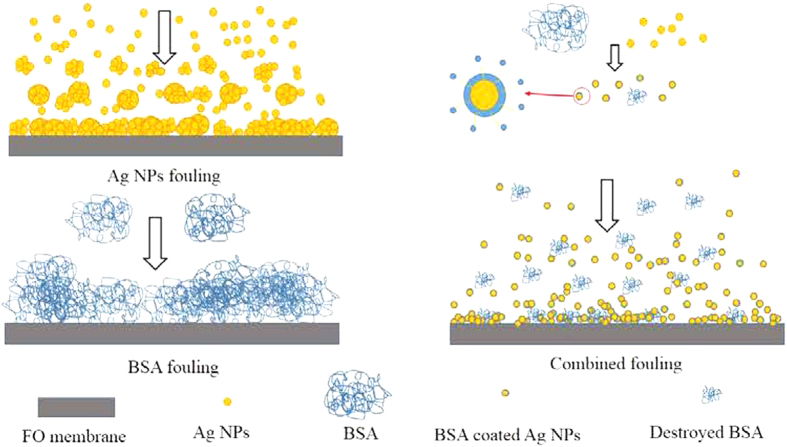
Illustration of the mechanisms for single and combined fouling of BSA and Ag NPs on FO membrane surface.

Based on the results obtained in current study, the single and combined FO membrane fouling of Ag NPs and BSA and the interactions between BSA and Ag NPs are schematically illustrated in Fig. 6. The special physical and chemical properties of nanoparticles such as quantum size effect, large specific surface area and high adsorption activity make the aggregation of Ag NPs easily occur^35^. During FO membrane filtration process, the concentration of Ag NPs nearby the membrane surface is higher than that in the bulk solution due to the concentrated external concentration polarization phenomenon^43^, which enhanced the possibility of nanoparticles collision, thus leading to the aggregation of Ag NPs and their deposition on FO membrane surface when single Ag NPs was present in feed solution. With regard to the single BSA fouling, the size of foulants deposited on FO membrane surface was much larger demonstrated by the SEM images in Fig. 2(c). In addition, when both BSA and Ag NPs are present in feed solution, protein macromolecules easily form nanoparticle-protein corona with a thickness of 3–7 nm surrounded nanoparticles^44^,^45^. The nanoparticle-protein corona can prevent nanoparticles from aggregation due to the steric stabilization mechanism^46^, which could explain the decrease of particle size deposited on FO membrane surface after adding BSA (see Fig. 2(a,d)). Meanwhile, it has been demonstrated that the addition of Ag NPs to the protein solution can destroy part of the α-helical secondary structure of the protein (see Fig. 4). This statement correlated well with the reduction of abundance and distribution of BSA on the FO membrane surface from the only BSA to both BSA and Ag NPs as the foulants (see Fig. 3). Thus, the interactions between Ag NPs and BSA both in solution and on membrane surface can alleviate the membrane fouling of Ag NPs to some extent depending on the concentration of Ag NPs.

## Materials and Methods

### Ag NPs and BSA

Commercially produced Ag NPs (99.9 wt% Ag, 20–40 nm, uncoated) purchased from Alfa Aesar were applied in this study. SDBS was chosen as an anionic surfactant to disperse the Ag NPs and enhance the stability. The stock suspension of Ag NPs (100 mg/L) was prepared by dispersing 0.1 g of raw Ag NPs in 1 L of SDBS water solution (0.1 wt%, pH 7.0), followed by 2 h of ultrasonication (25 °C, 500 W, 20 kHz) and agitation (100 r/min, 2 h) according to the related literature^47^ and then stored in a refrigerator at 4 °C.

BSA was selected as a model organic foulant to represent protein. Its molecular weight is about 66.4 kDa. The stock solutions of BSA (2 g/L) were prepared by dissolving solids in deionized (DI) water with continuous stirring at room temperature for over 24 h. Then, the BSA solution was transferred into a sterilized glass bottle and stored at 4 °C.

According to previous studies^23^,^48^, the typical concentrations of Ag NPs (1 mg/L) and BSA (200 mg/L) were selected in this study. The single Ag NPs and BSA feed solutions were obtained by directly diluting their stock solutions, while the combined feed solution of Ag NPs and BSA was prepared by mixing their diluted solution (1 and 200 mg/L, respectively) followed by ultrasonication (25 °C, 500 W, 20 kHz) and agitation (25 °C, 100 r/min) for 10 min to ensure complete dispersion of the Ag NPs. Average particle size and Zeta potential of single and combined suspensions were analyzed by a Zetasizer (Malvern Instruments, UK). The results (as shown in Table 1) indicated that the average particle size of mixed suspensions was almost similar to the single Ag NPs solution, but much less than the single BSA solution. Furthermore, the Zeta potential of the mixed solution was among the single Ag NPs and BSA solutions.

### FO membrane

The polyamide-based TFC-FO membranes with embedded polyester screen support were supplied by Hydration Technology Innovations (HTI, Albany, OR). The total thickness of the TFC-FO membrane is approximately 47 ± 3 μm, and its salt rejection is above 99.6% (FO mode, 1.0 M NaCl). Membrane samples were stored in DI water at 4 °C and then soaked in DI water at temperature-controlled room for 24 h before each experiment. The water permeability coefficients (*A*) and salt permeability coefficients (*B*) of both FO membranes were determined by reverse osmosis (RO) filtration tests at 8.62 bar as described by Cath *et al.*^49^. The *A* and *B* values were about 4.9 × 10^−12^ m/(s Pa) and 0.95 × 10^−7^ m/s, respectively. Its contact angel and Zeta potential at pH 6 are 45° ± 4° and −10 ± 5 mV, respectively^42^.

### Bench-scale FO experimental setup

**Figure 7:**
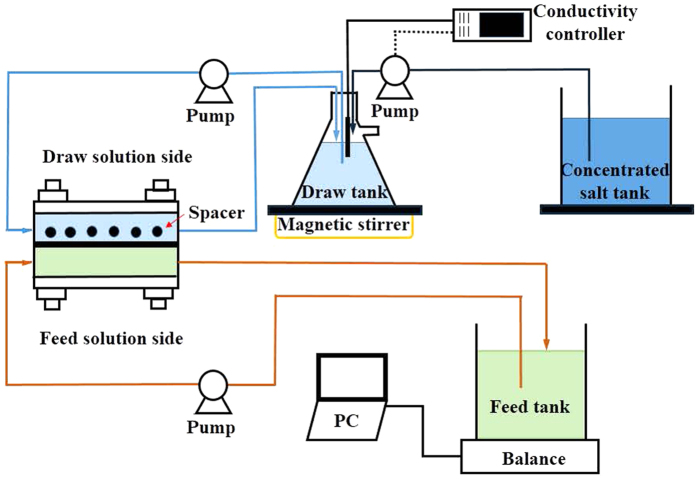
Schematic diagram of the bench-scale FO system.

A bench-scale FO cell with two channels was used to evaluate water flux and fouling behaviors of FO membrane at different operating conditions, as schematically shown in Fig. 7. The dimensions of both channels were 8.5 cm in length, 3.9 cm in width, and 0.2 cm in depth. The FO membrane with an effective area of 33.15 cm^2^ was placed in the middle of the FO cell between the two channels. The FO membrane orientation in the FO cell was also important for water flux and membrane fouling^50^,^51^. In current study, the active layer facing feed solution (AL-FS) was applied. It should be pointed out that a mesh spacer (pores size of 2.5 mm × 2.5 mm and a thickness of 0.5 mm) was applied in the draw solution side for alleviating the concentration polarization.

Two peristaltic pumps (Longer Pump WT600-2T) were used to recirculate the draw solution and feed solution, respectively. The feed solution tank was placed on a digital balance (Mettler Toledo ME-6002) and monitored by a computer with data acquisition software to record the permeate flux every 2 minutes. After a large number of preliminary experiments, a 5.0 M NaNO_3_ solution was used as the draw solution in all experiments because NaNO_3_ can not only avoid the precipitation of silver ions but also minimize ICP and create high osmotic pressures^52^. Furthermore, it can be easily regenerated by the RO process. The molar mass and density of NaNO_3_ are 84.9947 g/mol and 2.257 g/cm^3^, respectively. Its back diffusion is 0.2775 mmoles/(m^2^ s)^52^. A conductivity control system was used to control the draw solution at constant concentration of 5.0 M, which included a conductivity probe in draw solution tank, a control device and a concentrated salt adding system. When the concentration in the draw solution tank was less than 5.0 M, the probe would give a signal to the control device, and then a peristaltic pump was started to transfer the concentrated draw solution of 8.0 M NaNO_3_ to the tank until the draw solution recovered its initial concentration. All experiments worked at a temperature-controlled room to maintain the temperature in the range of 25 ± 0.5 °C.

### Protocols of fouling experiments

For each fouling test, a fresh FO membrane sample was soaked in DI water at 25 °C in a temperature-controlled room for 24 h, and then placed into the bench-scale FO test cell with the mode of AL-FS. Each membrane fouling experiment was conducted in the following steps: (1) stabilizing the system with DI water for 1 h to remove the impurities from membrane surface; (2) testing the virgin membrane flux with fresh DI water for 3–4 h until reach a stable water flux of 18.0 ± 0.5 LMH at a cross-flow velocity of 12.9 cm/s and (3) continuously performing the fouling experiment with freshly prepared feed solution and draw solution for 11 h at a cross-flow velocity of 12.9 cm/s.

### Calculations of concentration factor and permeat fraction

The *CF*_*w*_ and the fraction of wastewater permeat out (*PF*) in the FO process can be determined by the following equations, respectively.


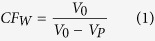



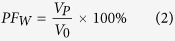


where *V*_*0*_ is the initial volume of feed wastewater (L) and *V*_*p*_ is the volume of the wastewater permeat out by FO membrane (L).

### Analytical methods

At the end of each experiment, the fouled membrane coupon was removed from the FO cell and dissected into some subsamples for CLSM, SEM and ATR-FTIR analyses.

According to a previous study^53^,^54^, FITC was used to label proteins. A central section from the fouled membrane was cut into 0.5 cm × 0.5 cm coupon, and then the FITC solution (10.0 g/L) was dripped onto the fouled membrane sample after 1 M NaHCO_3_ buffer was applied for keeping the amine group in a non-protonated form. After the labeling process, the sample was incubated for 30 min at room temperature in the dark, and then was washed twice with phosphate buffered saline (PBS) solution to remove the extra probes. The stained FO membrane samples were characterized by CLSM (LEICA TCS SP5, Germany) with the excitation and emission wavelengths of 48 nm and the range of 500–540 nm, respectively. Three dimensional reconstructions were obtained with LASAFE confocal software.

The morphology of membrane surface was characterized with SEM (Hitachi S4800, Japan) at 15 kV, and the chemistry and elemental composition of fouled membrane were determined by ATR–FTIR on a spectrophotometer (Nicolet IS50, Thermo-Fisher, USA). Prior to SEM and ATR–FTIR observations, all FO membrane samples were prepared by freezing the membrane at −80 °C in a chiller for 2 h followed by freeze drying at −48 °C for 6 h using a freeze dryer (Free Zone 25, Labconco, Czech Republic).

## Additional Information

**How to cite this article**: Zhao, Y. *et al.* Nanoparticle fouling and its combination with organic fouling during forward osmosis process for silver nanoparticles removal from simulated wastewater. *Sci. Rep.* **6**, 25859; doi: 10.1038/srep25859 (2016).

## Figures and Tables

**Table 1:** Characteristics of feed solution.

Feed solute	Single Ag NPs	Single BSA	Ag NPs & BSA
Concentration (mg/L)	1	200	1&200
Average particle diameter (nm)	123.5 ± 0.3^a^	432.6 ± 2.5^a^	124.3 ± 0.2^a^
Zeta potential (mV)	−0.267 ± 0.002^a^	0.884 ± 0.004^a^	0.263 ± 0.003^a^

^a^Values are given as mean values ± standard deviation (number of measurements: n = 3).
